# Lithospheric delamination and upwelling asthenosphere in the Longmenshan area: insight from teleseismic P-wave tomography

**DOI:** 10.1038/s41598-019-43476-0

**Published:** 2019-05-06

**Authors:** Chuansong He, Shuwen Dong, Yanghua Wang

**Affiliations:** 1 Institute of Geophysics, CEA, Beijing, 100081 China; 2 State Key Laboratory for Mineral Deposits Research, Nanjing University, Nanjing, 210093 China; 3 Department of Earth Science and Engineering, Imperial College London, South Kensington, London SW7 2BP UK

**Keywords:** Geophysics, Seismology, Tectonics

## Abstract

We apply teleseismic P-wave tomography to reconstruct the velocity structure of the Longmenshan area. Our results show possible large-scale delamination beneath the Songpan-Ganzi and Qiangtang terranes, which induced upwelling asthenosphere. Upwelling asthenosphere might have led to lower crust heating, facilitating eastward extrusion of the Songpan Ganzi terrane resulting in localized deformation and uplift along the Longmenshan orogenic belt. We suggest that the eastward extrusion of the Songpan-Ganzi terrane against the rigid lithospheric root of the Sichuan Basin results in stress accumulation and release, leading to large earthquakes in the Longmenshan area.

## Introduction

**Figure 1:**
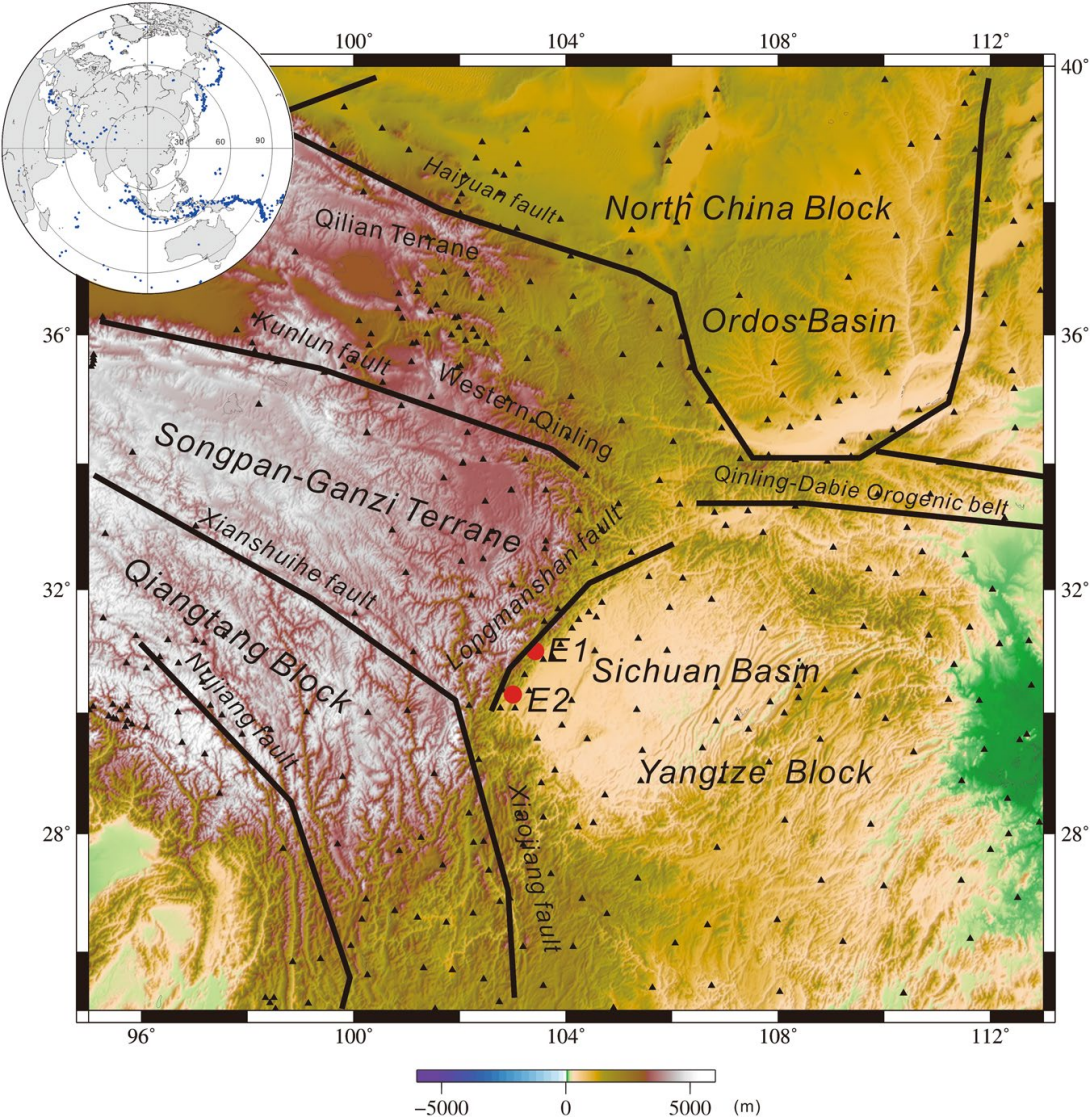
Tectonic framework and distribution of seismic stations (black triangles). Inset figure: events with epicentral distances ranging from 30°–85° and earthquake magnitudes >6.0, which are used by this study. E1: 2008 Mw 7.9 Wenchuan earthquake. E2: 2013 Mw 6.6 Lushan earthquake (The figure was generated by Chuansong He using the Generic Mapping Tool (http://gmt.soest.hawaii.edu/)).

The Longmenshan thrust (or orogenic) belt is approximately 500 km long and 30–50 km wide^1–7^ with a relief of over 5 km^8^. It is the central segment of the north-south-trending major seismic belt in China and lies along a crustal-scale boundary between the younger and possibly weaker Triassic Songpan-Ganzi terrane in the northwest and the stronger lithospheric root of the Yangtze block to the south^9,10^ (Fig. 1).

The deformation and uplift of the Longmenshan orogenic belt were initiated as early as the Middle to Late Triassic and were associated with the Indosinian orogeny and the amalgamation of the North China, South China and Qiangtang continental blocks^11–13^. Reactivation during Cenozoic Indian-Asian collision resulted in the formation of one of the steepest continental escarpments in the world^14–16^. The Longmenshan orogenic belt has been a key region to evaluate the Mesozoic-Cenozoic tectonic evolution of China^17–21^ and has also been termed the “Asian puzzle”^22^ with important records of continental collision, magmatism, basin evolution and earthquakes^23^.

The critical tectonic location and frequent earthquakes have made the Longmenshan area the focus of various investigations such as petrology, GPS, numerical simulation, geochronology, geomorphology and geophysics in the last several years^15,21,24–28^. However, the deformation and uplift mechanism for the Longmenshan orogenic belt remains controversial, and two end-member models have been proposed: (1) Crustal thickening through major slip along thrust faults rooted in the lithosphere^29–31^ and (2) Channel flow or extrusion of partially molten middle- to lower-crustal materials with extremely low viscosity outwards from the Tibetan Plateau since approximately 40 Ma^4,10,14,25,32,33^. Obviously, these works do not take into account the effect of mantle dynamics.

To decipher the crustal and upper mantle structure and investigate the deformation and uplift mechanism of the Longmenshan orogenic belt, multi-disciplinary geophysical studies have been carried out, including deep seismic sounding^34–40^, receiver functions^32,41–43^, magnetotelluric surveys^44^, and tomography^45–50^. However, most of these studies have been broadly related to the velocity structure and tectonic architecture. The dynamic and tectonic evolution of this belt remains enigmatic^2,51–56^.

In this work, we collected high-quality seismic data recorded by the China earthquake network and employed teleseismic P-wave tomography to detect the upper mantle velocity structure. The results show three high-velocity perturbations under the Songpan-Ganzi terrane, Ordos Basin and Sichuan Basin. These high-velocity structures are at almost the same depth of ca. 400–500 km, and we suggest that these high-velocity structures may be attributed to ancient lithosphere that could have been delaminated beneath the Songpan-Ganzi terrane, Ordos Basin and Sichuan Basin. Specifically, a large-scale low-velocity perturbation covers almost all the Songpan-Ganzi area in the uppermost mantle; under this anomaly, there is a high-velocity anomaly (plate-like) at 400–600 km depth, which might be the lithosphere of the Songpan-Ganzi terrane that was completely removed, recycled, and accumulated into the upper mantle. The lower crust of the Songpan-Ganzi terrane directly contacts the hot asthenospheric mantle (low-velocity perturbation). We propose that asthenosphic upwelling heated the lower crust, facilitating the easy eastward extrusion of the Songpan-Ganzi terrane and resulting in uplift and deformation of the Longmenshan orogenic belt in the Cenozoic due to the Indian-Asian plate collision. The rigid lithosphere of the Sichuan Basin obstructs the eastward extrusion of the Songpan-Ganzi terrane, which causes stress accumulation and release in the Longmenshan area and produces large earthquakes in this region^14,25^.

## Results

**Figure 2:**
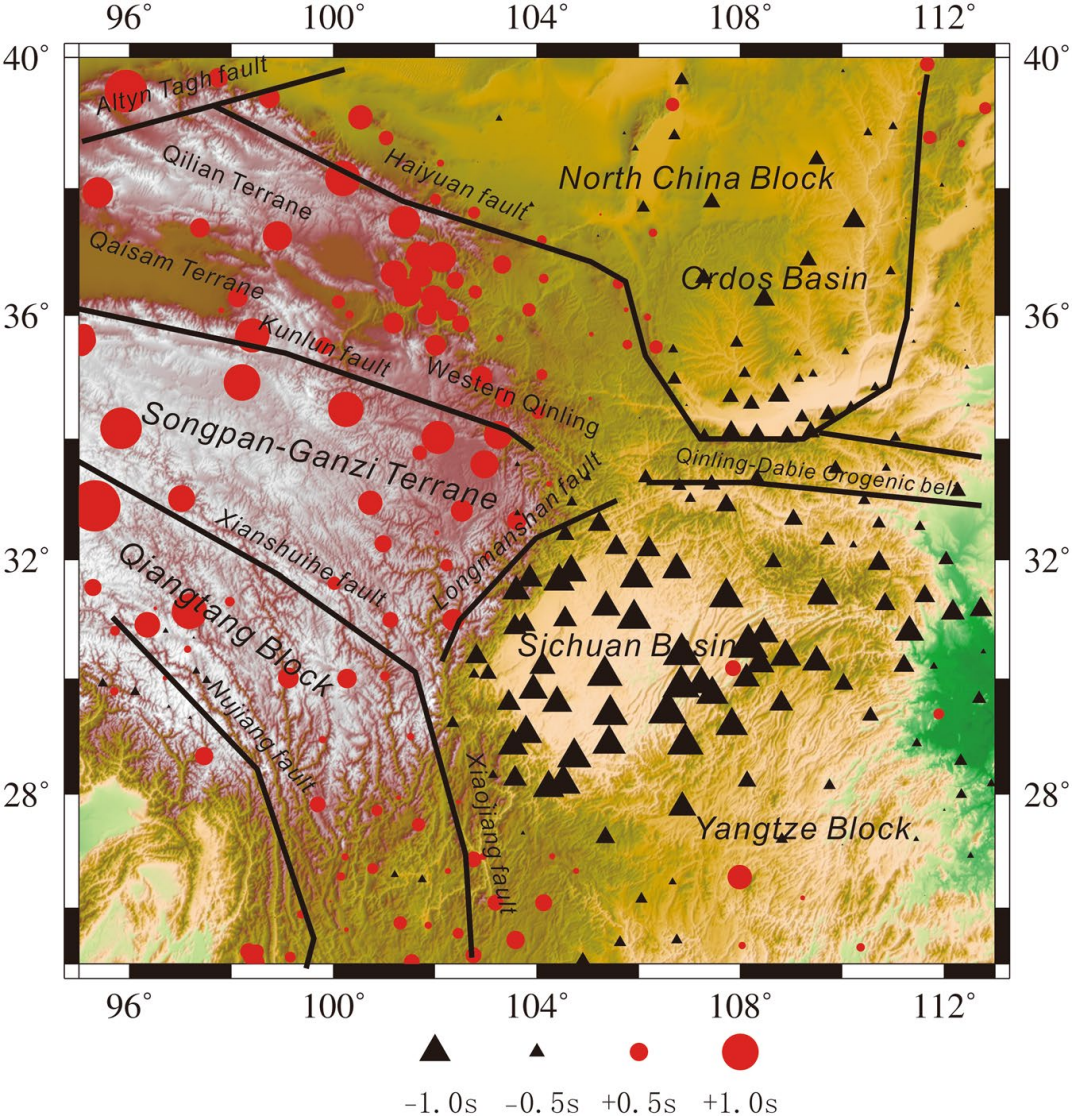
Average value of the relative travel-time residual at each station. Black triangles: high-velocity region; red circles: low-velocity region (The figure was generated by Chuansong He using the Generic Mapping Tool (http://gmt.soest.hawaii.edu/)).

The average value of the relative travel-time residual at each station is negative in the western part of the study region or the Longmenshan orogenic belt and positive in the eastern part of the study region (Fig. 2), which suggests the presence of lower- and higher-velocity anomalies beneath the western and eastern parts of the Longmenshan orogenic belt, respectively.

**Figure 3:**
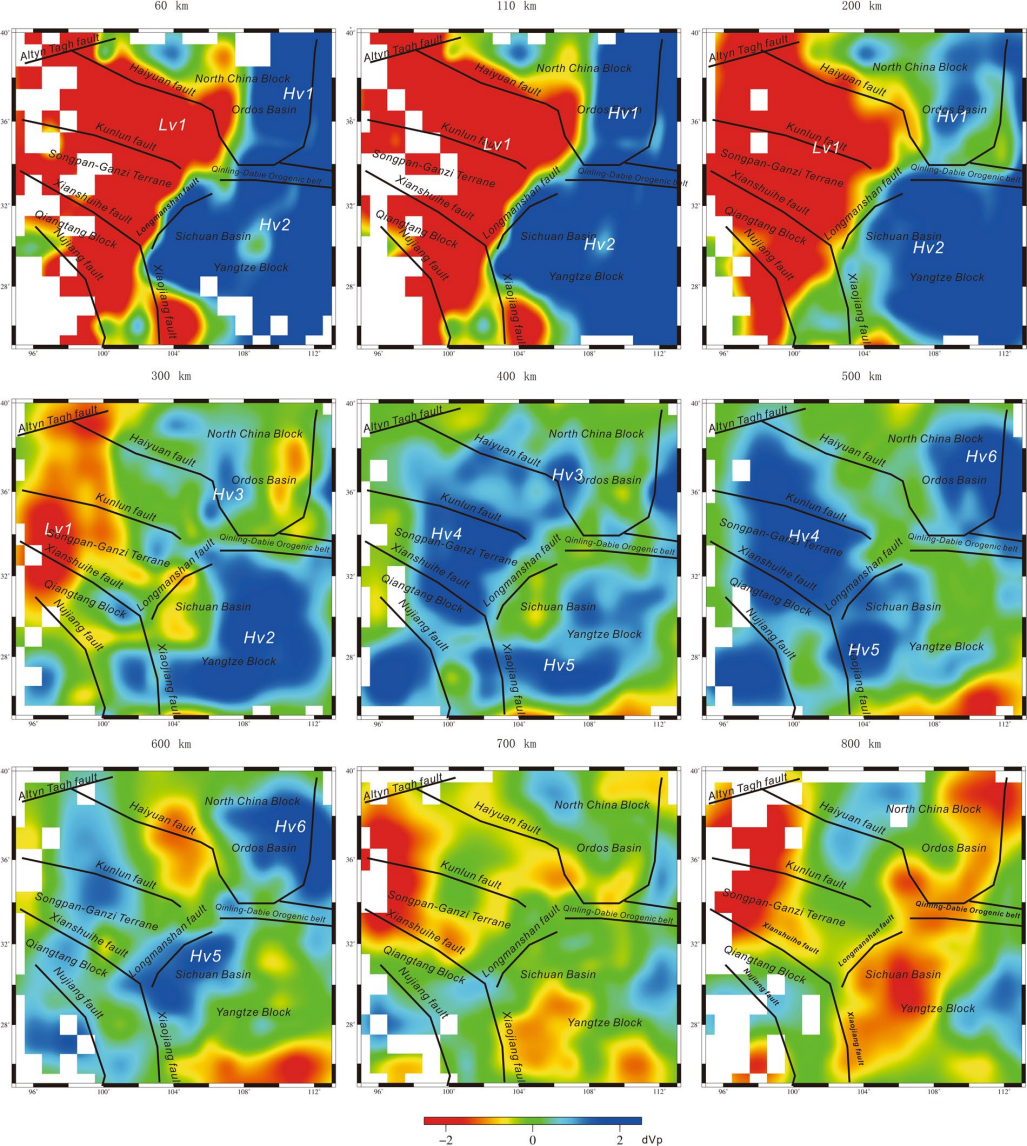
P-wave velocity perturbations at depths of 60, 110, 200, 300, 400, 500, 600, 700 and 800 km. Portions of the model where the recovery of the starting model in the CRT was below 20% are not shown (see Fig. S3) (The figure was generated by Chuansong He using the Generic Mapping Tool (http://gmt.soest.hawaii.edu/)).

The 60, 110, 200 and 300 km depths reveal a prominent and large-scale low-velocity perturbation (Lv1) in the western part of the Longmenshan orogenic belt (Fig. 3). Huang *et al*.^57^ and Yang *et al*.^58^ also determined similar results. Adjoint tomography^59^, ambient noise tomography^60^, East Asia mantle tomography^61^ and multi-scale travel-time tomography^48^ also show a low-velocity structure beneath the Songpan-Ganzi terrane. In the eastern part of this study area, a large-scale high-velocity perturbation is observed at 60, 110 and 200 km depths beneath the Ordos Basin (Hv1), and another large-scale high-velocity perturbation is observed at 60, 110, 200 and 300 km beneath the Sichuan Basin (Hv2) (Fig. 3), which is consistent with previous studies^57,58,62–64^. Xin *et al*.^65^ also indicated high-velocity structures beneath the Sichuan and Ordos Basins using double-difference seismic travel-time tomography. Hv1 and Hv2 should be the lithospheric roots of the Ordos Basin and Sichuan Basin, respectively.

At 300 and 400 km depths, there is a small high-velocity perturbation (Hv3) (Fig. 3), which is located within the Ordos Basin. At 400, 500 and 600 km depths, there are three high-velocity anomalies (Hv4, Hv5 and Hv6) beneath the western part of the Longmenshan orogenic belt, the Sichuan Basin and the Ordos Basin (Fig. 3), respectively. Due to the low resolution, we discard the features on the 700 and 800 km depth sections.

**Figure 4:**
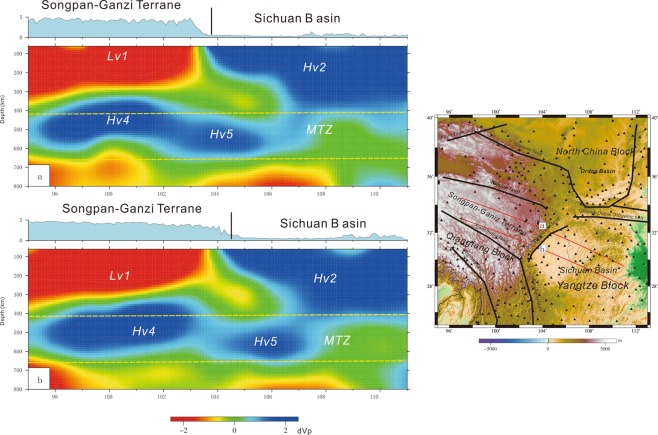
Profiles a and b of velocity perturbations (The figure was generated by Chuansong HE using the Generic Mapping Tool (http://gmt.soest.hawaii.edu/)). MTZ: Mantle transition zone. Vertical line: Longmenshan fault.

**Figure 5:**
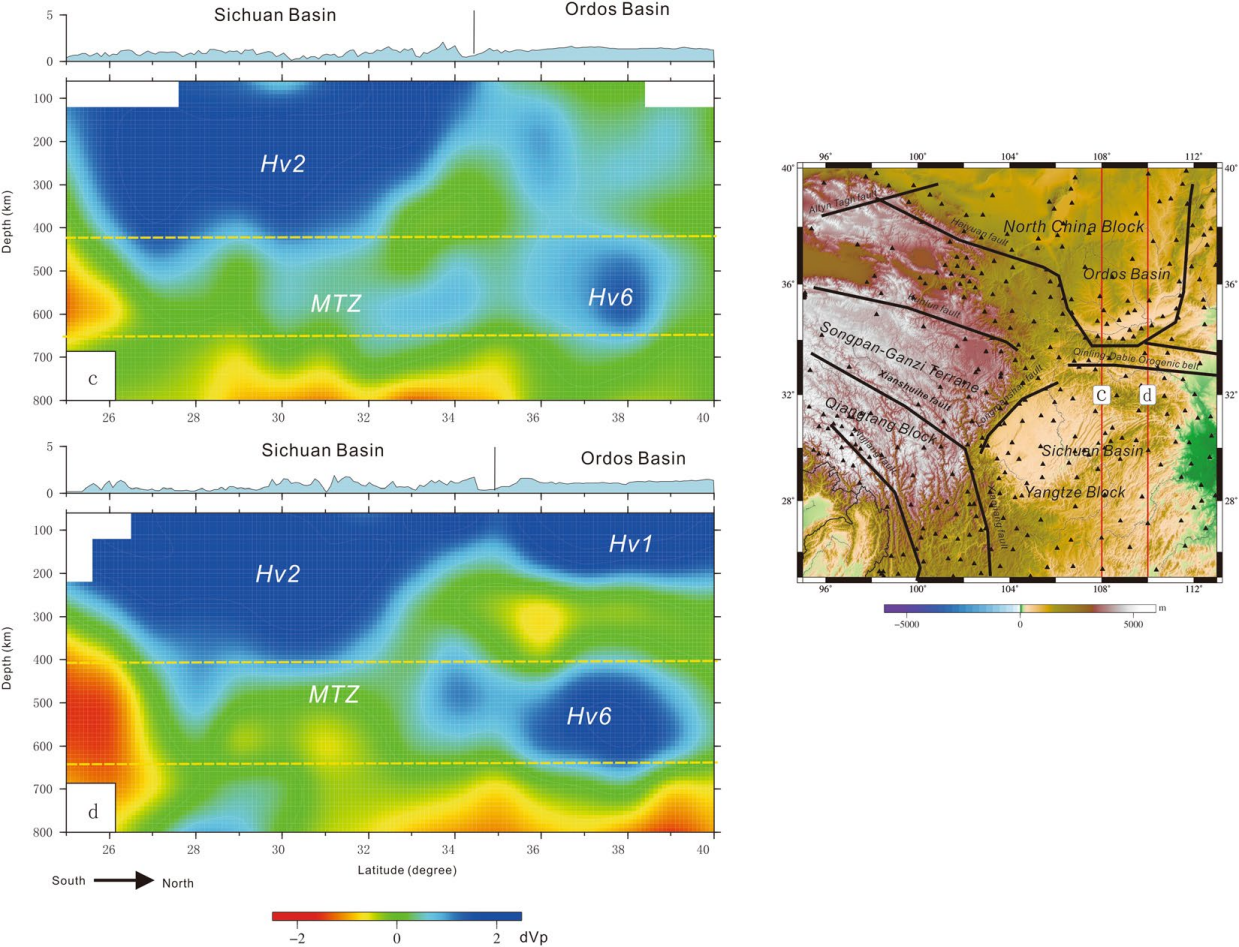
Profiles c and d of velocity perturbations. Portions of the model where the recovery of the starting model in the CRT was below 20% are not shown (The figure was generated by Chuansong He using the Generic Mapping Tool (http://gmt.soest.hawaii.edu/)). MTZ: Mantle transition zone.

Profiles a and b show that Lv1 is approximately 300 km thick and Hv2 is approximately 350 km thick (Fig. 4a,b). A larger high-velocity structure (Hv4) lies beneath the Songpan-Ganzi terrane, and another high-velocity structure (Hv5) underlies the Sichuan Basin (Fig. 4). Profiles c and d show approximately 350 km lithospheric thickness in the Sichuan Basin and approximately 200 km lithospheric thickness in the Ordos Basin (Fig. 5), and there is a high-velocity structure (Hv6) beneath the Ordos Basin (Fig. 5).

Huang *et al*.^57^ also revealed two high-velocity structures beneath the south part of the Songpan-Ganzi terrane and the Sichuan Basin at 450–600 km depths, but these depths and the locations of the high-velocity structures are different from our results. Yang *et al*.^58^ showed a high-velocity structure beneath the Sichuan Basin at 300–500 km depths, which is similar to our Hv5 (Figs 3 and 4).

However, most studies did not reveal the high-velocity structures Hv4 beneath the Songpan-Ganzi terrane and Hv6 beneath the Ordos Basin. Moreover, three high-velocity structures (Hv4, Hv5 and Hv6) identified by this study are at the same depth (Figs 4 and 5), and, there is an especially good correspondence between the high-velocity (Hv4) and low-velocity (Lv1) perturbations in the Songpan-Ganzi terrane (Fig. 4). Our results also indicate the Longmenshan orogenic belt is the boundary between the low- and high-velocity perturbations (Fig. 4).

## Discussion

### Lithospheric delamination

The Indosinian orogeny might have imposed its imprints upon the seismic velocity structure and composition in the crust and upper mantle^66^. A number of studies have demonstrated that the high- and low-velocity relics generated by lower crustal/lithospheric delamination (or a subducting slab) and upwelling asthenosphere can be retained for two billion years and can be detected by seismic techniques^67–74^. The large-scale high-velocity perturbation (Hv4) beneath the Songpan-Ganzi and Qiantang terranes (Figs 3 and 4) is approximately 200 km thick, which is thicker than the crust; accordingly, we suggest this feature might be associated with the delamination of the thickened lower crust/lithosphere mantle. This region has been affected by two major tectonic events: one is the Indosinian orogeny in the Early Triassic, and the other is the Himalayan orogeny in the Cenozoic. Based on the scale and depth of Hv4, we speculate it might either have been induced by the collision and amalgamation among the Yangtze, North China and Qiangtang terranes during the Indosinian orogeny in the Mesozoic or during the Cenozoic India-Asia collision. Previous interpretations of tomographic models have proposed the lithosphere foundering of lithosperic slabs beneath the Tibetan Plateau due to the northward subduction of the Indian Plate during the Himalayan orogeny in the Cenozoic^75^. Therefore, we cannot exclude that the lithosphere mantle of the Songpan-Ganzi terrane may have been removed by the eastward extrusion of the Tibetan Plateau or that the high-velocity structure (Hv4) may be linked to the subduction of the lithosphere in the Cenozoic^76,77^.

Orogenies generally lead to lithospheric delamination simultaneously or after collision^78^. Geological studies demonstrated that during the Early Triassic Indosinian orogeny, the crust of the Songpan-Ganzi terrane was strongly thickened through continental collision among the Yangtze, the North China and the Qiangtang blocks^53–55,79–83^, which accompanied the closure of the Paleotethys Ocean^84^. The thickened lower crust experienced eclogite-facies metamorphism, leading to increased density that might have resulted in the delamination of the lower crust/lithosphere in the Songpan-Ganzi terrane in the Middle Triassic^79^.

Delamination can result in upwelling asthenosphere that fills voids formed by delamination^85^. Just above Hv4 is a large-scale low-velocity perturbation (Lv1); we therefore suggest that this large-scale low-velocity structure may have been produced by upwelling asthenosphere that filled the voids generated by delamination. Petrological studies have demonstrated that in the Songpan-Ganzi and Longmenshan terranes, magmatic events are recorded throughout the Mesozoic^81–83,86^, which should have been associated with the upwelling asthenosphere. This upwelling asthenosphere might have heated the base of the lower crust and promoted partial melting to generate adakitic magmas in the lower crust and/or A-type granitic magma^79^. Geochemical studies show that the adakitic and A-type granite magmatism in the Songpan-Ganzi and Longmenshan terranes occurred during the Late Triassic within a post-collisional setting^79^.

On the other hand, Hv5 and Hv6 (as well as Hv3) are under the Sichuan Basin and Ordos Basin, respectively. The root of the Sichuan Basin lithosphere is thicker than that of the Ordos Basin; however, the scale of Hv6 is larger than that of Hv5 (Figs 4 and 5). Based on this contrast, we suggest that lithospheric delamination to different degrees occurred beneath the Sichuan Basin and the Ordos Basin in the Late Triassic due to the stronger collision among the Qiangtang, Yangtze and North China blocks^86^. Hv5 might have been part of the Sichuan Basin lithosphere, and Hv6 might have been part of the Ordos Basin lithosphere in the Early Triassic.

### Deformation and uplift mechanism of the longmenshan orogenic belt

The channel flow model of the middle/lower crust^32,43,87–89^ has been employed in some studies to explain the formation of the Longmenshan orogenic belt. Low to moderate Poisson’s (or Vp/Vs) ratios in the Songpan-Ganzi and Longmenshan area suggest that the lower crust is dominated by felsic to intermediate compositions^41,90^, which can preserve partial melting 20 to 30 Ma after crustal thickening^91,92^. Geophysical studies indicate the low-velocity zone associated with high conductivity at a depth below 10 km on the southern Tibetan Plateau^93^. Therefore, we cannot exclude the possibility that the crustal thickening was generated by the channel flow of the middle/lower crust.

Furthermore, many workers believe that the crustal thickening that occurred in the Longmenshan area was caused by ductile deformation rather than by thrust faulting or crustal shortening^4,24^. The eastward extrusion of the Tibetan Plateau is considered associated with rapid slip along these faults^94^, and significant relative motion along major strike-slip faults facilitated the eastward extrusion of crustal material out of the Tibetan Plateau^95–97^.

Our study shows that the inferred large-scale delamination beneath the Songpan-Ganzi and Qiangtang terranes resulted in the removal of more rigid lithosphere and the upwelling asthenosphere, leading to further heating of the lower crust and producing ductile and easily deformed lower crust or forming channel flow of the middle/lower crust. Moreover, the upwelling asthenosphere directly contacted the lower crust, resulting in a detachment surface between the lower crust and upper mantle, facilitating the easy eastward extrusion of the Songpan-Ganzi terrane and leading to the ductile deformation or thickening of the lower crust of the Songpan-Ganzi terrane due to the collision between the Indian and Eurasian plates in the Cenozoic.

The large-scale high-velocity perturbation beneath the Sichuan Basin (Hv2) (Figs 3–5) is the old, strong lithosphere that underlies the Sichuan Basin^31,46,98^, pushing up the crust above and achieving steep topography through dynamic pressure. This process accounts for the deformation and uplift of the Longmenshan orogenic belt.

### Earthquakes

A ductile crust plays an important role in generating earthquakes through coseismic slip and rupture along the Longmenshan fault zone. The lower-crustal material flowing outward from the Songpan-Ganzi block might have been supported by a strong craton-like lithosphere underlying the Sichuan foreland basin^46^. The present-day strain energy related to continental deformation in the central Tibetan Plateau, generated through collision between the Indian and Eurasian plates, is related to strike-slip faulting along active strike-slip faults such as the Kunlun and Xianshuihe faults, generating strong earthquakes that release the accumulated strain energy^99^.

The surface ruptures of the 1997 Manyi, 2001 Kokoxili, and 2010 Yushu earthquakes were dominated by pure left-lateral faulting along the WNW-trending strike-slip faults, representing an eastward motion of the Songpan-Ganzi and Qiangtang blocks bounded by mega-strike-slip faults in the northern Tibetan Plateau^95,99–103^.

Meanwhile, eastward movement of the Songpan-Ganzi block against the lithospheric root of the Sichuan Basin (Hv2) (Fig. 4) results in large stress accumulation along the Longmenshan fault zone. When the stress exceeds the threshold, it is suddenly released through a violent rupture along the faults. The Longmenshan area is likely indicative of cumulative offsets from earthquakes^4,28,104^. Our results support the notion that the eastward extrusion of the Songpan-Ganzi block might have resulted in stress concentration mostly at the bottom of the Longmenshan fault zone, which is thought to be responsible for the generation of the 2008 Mw 7.9 Wenchuan and 2013 Mw 6.6 Lushan earthquakes (Fig. 1: E1 and E2). Therefore, we suggest that the northward movement of the Indian plate entrains the eastward extrusion of the Songpan-Ganzi terrane, causing stress accumulations and releases as well as large earthquakes in the Longmenshan area.

## Conclusions

Our results suggest that there was massive delamination of the lower crust/part of the lithosphere in this area during the Late Triassic due to the stronger collision and amalgamation of the North China, South China and Qiangtang continental blocks. We cannot exclude the notion that the large-scale high-velocity structure beneath the Songpan-Ganzi terrane may be linked to the subduction of the lithosphere or the removal of part of the rigid lithosphere in the Cenozoic due to the eastward extrusion of the Tibetan Plateau induced by the Indian-Asian collision. The large-scale delamination of the lithosphere resulted in upwelling asthenosphere, which enhanced the ductile nature of the Songpan-Ganzi block, resulting in crustal thickening, deformation and uplift of the Longmenshan belt in the Cenozoic due to the eastward extrusion of the Tibetan Plateau. The eastward extrusion of the Songpan-Ganzi block is inhibited by the rigid lithosphere of the Sichuan Basin, generating stress accumulation in the Longmenshan area. When the stress exceeds the threshold, its sudden release along the faults accounts for the violent earthquakes in the Longmenshan region, such as the 2008 Mw 7.9 Wenchuan earthquake and the 2013 Mw 6.6 Lushan earthquake.

## Data and Method

For this study, we collected 554 teleseismic events recorded by Chinese earthquake networks with 403 seismic stations from July 2007 to March 2014 (Fig. 1)^105^. The seismic events with magnitudes >6.0 and epicentral distances ranging from 30°–85° were selected (Fig. 1, insert figure). Waveforms were cut 15 s before and 50 s after the first P-wave arrival from digital seismograms and filtered between 0.3 and 3 Hz. We selected 66492 first P-wave arrivals from the cut and filtered seismograms of seismic events using the time cross-correlation method^106^ (Fig. S1). The range of −2.5 s to +2.5 s in the relative travel-time residuals was used in the tomographic inversion (Fig. S2).

The lateral grid interval was 1° in this optimal Vp model, and vertical grid intervals were designed at 60, 110, 200, 300, 400, 500, 600, 700 and 800 km depths. The computation of ray paths and theoretical travel times was performed by an efficient 3-D ray-tracing method^72^. We adopted a conjugate-gradient inversion algorithm^107^ with smoothing and damping regularizations to determine the sparse and large system of observation equations^72^. In the 3-D tomographic inversion, the iasp91 1-D Earth model^108^ was used as the starting model.

The correction of the relative travel-time residuals was done with the CRUST1.0 model^109^ following the method of Jiang *et al*.^110^, which removed the effect of crustal heterogeneity generated by crustal thickness and velocity^110,111^. Based on the upper depth limit of tomography, we applied a crustal correction for the upper 60 km of the lithosphere. The results showed that the corrected value induced a large effect on the distribution of the relative travel-time residuals (Figs S3 and S4), and the larger corrected values were mainly distributed in the western part of the study region (Fig. 4). To select a suitable damping parameter for use in the tomographic inversion, we performed numerous inversions with different damping parameters to derive a trade-off or L-shaped curve that could measure the misfit of each solution model with the data^112–114^. Eventually, a damping value of 11.0 was taken to invert the final solution model (Fig. S5).

The checkerboard resolution test (CRT) was employed to assess the reliability of the obtained tomographic images and the adequacy of ray coverage. All the grid nodes of the 3-D space were assigned 2.5% negative and positive velocity perturbations. Figure S6 shows the CRT result. In general, most parts of the study region have good resolution (Fig. S6), where the checkerboard pattern and the amplitude of velocity anomalies are well recovered. The resolution is reduced at 60, 700 and 800 km depths and in the western part of the study area, indicating that the seismic rays do not crisscross well in these locations.

## Supplementary information

Supplementary Information

## References

[1] Deng, Q. D., Chen, S. F. & Zhao, X. L. Tectonics, seismisity and dynamics of Longmen Shan and its adjacent regions. *Seis*. *Geol*. **16**, 404–421 (in Chinese with English abstract) (1994).

[2] Li Y, Allen PA, Densmore AL, Xu Q (2003). Evolution of the Longmen Shan foreland basin (western Sichuan, China) during the late Triassic Indosinian orogeny. Basin Res..

[3] Burchfiel BC (2004). New technology, new geological challenges. GSA Today.

[4] Burchfiel BC (2008). A geological and geophysical context for the Wenchuan earthquake of 12 May 2008, Sichuan, People’s Republic of China. GSA Today.

[5] Kirby E, Whipple KX, Harkins N (2008). Topography reveals seismic hazard. Nat. Geosci..

[6] Godard V (2009). Late Cenozoic evolution of the central Longmen Shan, eastern Tibet: insight from (UTh)/He thermochronometry. Tectonics.

[7] Li ZG, Jia D, Chen W (2013). Structural geometry and deformation mechanism of the Longquan anticline in the Longmen Shan fold-and-thrust belt, eastern Tibet. J. Asian Earth Sci..

[8] de Michele M, Raucoules D, de Sigoyer J, Pubellier M, Chamot-Rooke N (2010). Three-dimensional surface displacement of the 2008 May 12 Sichuan earthquake (China) derived from Synthetic Aperture Radar: evidence for rupture on a blind thrust. Geophys. J. Inter..

[9] Yin An (2010). A special issue on the great 12 May 2008 Wenchuan earthquake (Mw7.9): Observations and unanswered questions. Tectonophysics.

[10] Sun S (2012). Seismic properties of the Longmen Shan complex: Implications for the moment magnitude of the great 2008 Wenchuan earthquake in China. Tectonophysics.

[11] Liu, S. G. *et al*. 4-D textural and structural characteristics of Longmen intracontinental composite orogenic belt, southwest China. *Chin*. *J*. *Geo*. **44**, 1151–1179 (in Chinese with English abstract) (2009).

[12] Chen FS, Wilson CJL (1996). Emplacement of the Longmen Shan Thrust-Nappe Belt along the eastern margin of Tibetan Plateau. J. Struct. Geol..

[13] Roger F, Jolivet M, Malavieille J (2010). The tectonic evolution of the Songpan Garzê (North Tibet) and adjacent areas from Proterozoic to Present: a synthesis. J. Asian Earth Sci..

[14] Clark MK, Royden LH (2000). Topographic ooze: building the eastern margin of Tibet by lower crustal flow. Geology.

[15] Kirby E (2002). Late Cenozoic evolution of the eastern margin of the Tibetan Plateau: inferences from 40Ar/+r and (U–Th)/He thermochronology. Tectonics.

[16] Li ZW (2012). Spatial variation in Meso-Cenozoic exhumation history of the Longmen Shan thrust belt (eastern Tibetan Plateau) and the adjacent western Sichuan basin: Constraints from fission track thermochronology. J. Asian Earth Sci..

[17] Sengör AMC, Hsü KJ (1984). The Cimmerides of eastern. Asia: history of the eastern end of Paleo-Tethys. Mem. Soc. Geol. Fr..

[18] Mattauer M (1985). Tectonics of the Qinling belt: build-up and evolution of eastern Asia. Nature.

[19] Xu, Z., Hou, Q. & Wang, Z. Orogenic processes of the Songpan-Garzê orogenic belt of China (eds Xu, Z., Hou, Q. & Wang, Z.) 190 (Geological Publishing House, Beijing 1992).

[20] Yin, A., Harrison, M. The Tectonic Evolution of Asia (eds Yin, A., Nie, S.) 442–485 (Cambridge University Press 1996).

[21] Jia D (2010). Structural model of 2008 Mw 7.9 Wenchuan earthquake in the rejuvenated Longmen Shan thrust belt, China. Tectonophysics.

[22] Enkin R, Yang Z, Chen Y, Courtillot V (1992). Paleomagnetic constraints on the geodynamic history of the major blocks of China from the Permian to the present. J. Geophys. Res..

[23] Xiao L (2007). Late Triassic granitoids of the eastern margin of the Tibetan Plateau: Geochronology, petrogenesis and implications for tectonic evolution. Lithos.

[24] Burchfiel BC, Chen Z, Liu Y, Royden LH (1995). Tectonics of the Longmen Shan and adjacent regions, Central China. Int. Geol. Rev..

[25] Royden LH (1997). Surface deformation and lower crustal flow in eastern Tibet. Science.

[26] Clark MK (2004). Surface uplift, tectonics, and erosion of Eastern Tibet from large-scale drainage patterns. Tectonics.

[27] Godard V (2010). Spatial distribution of denudation in Eastern Tibet and regressive erosion of plateau margins. Tectonophysics.

[28] Liu C, Zhu BJ, Yang XL, Shi YL (2015). Crustal rheology control on earthquake activity across the eastern margin of the Tibetan Plateau: Insights from numerical modelling. J. Asian Earth Sci..

[29] Tapponnier P (2001). Oblique stepwise rise and growth of the Tibet Plateau. Science.

[30] Xu ZQ (2008). Uplift of the Longmen Shan range and the Wenchuan earthquake. Episodes.

[31] Hubbard J, Shaw J (2009). Uplift of the Longmen Shan and Tibetan plateau, and the 2008 Wenchuan (M = 7.9) earthquake. Nature.

[32] Zhang ZJ (2009). Crustal structure across Longmenshan fault belt from passive source seismic profiling. Geophys. Res. Lett..

[33] Vanderhaeghe O, Teyssier C (2001). Crustal-scale rheological transitions during late-orogenic collapse. Tectonophysics.

[34] Wang YX, Mooney WD, Han GH, Xu CY, Jiang M (2005). The crustal P-wave velocity structure from Altyn Tagh to Longmen Mountains along the Taiwan-Altay geoscience transection. Chin. J. Geophys..

[35] Liu M, Mooney WD, Li S, Okaya N, Detweiler S (2006). Crustal structure of the northeastern margin of the Tibetan plateau from the Songpan-Ganzi terrane to the Ordos basin. Tectonophysics.

[36] Zhang Z, Klemperer S, Bai Z, Chen Y, Teng J (2011). Crustal structure of the Paleozoic Kunlun orogeny from an active-source seismic profile between Moba and Guide in East Tibet, China. Gondwana Res..

[37] Zhang Z (2013). Crustal structure across northeastern Tibet from wide-angle seismic profiling: Constraints on the Caledonian Qilian orogeny and its reactivation. Tectonophysics.

[38] Gao R (2014). The crust structures and the connection of the Songpan block and est Qinling orogen revealed by the Hezuo-Tangke deep seismic reflection profiling. Tectonophysics.

[39] Lu R (2014). Structural model of the central Longmen Shan thrusts using seismic reflection profiles: Implications for the sediments and deformations since the Mesozoic. Tectonophysics.

[40] Lu R, He D, Xu X, Liu B (2016). Crustal-scale tectonic wedging in the central Longmen Shan: Constraints on the uplift mechanism in the southeastern margin of the Tibetan Plateau. J. Asian Earth Sci..

[41] He CS, Dong SW, Santosh M, Chen XH (2014). Seismic structure of the Longmenshan area in SW China inferred from receiver function analysis: Implications for future large earthquakes. J. Asian Earth Sci..

[42] Li X, Santosh M, Cheng S, Xu X, Zhong W (2015). Crustal structure and composition beneath the northeastern Tibetan plateau from receiver function analysis. Phys. Earth Planet. Inter..

[43] Sun Y, Liu J, Zhou K, Chen B, Guo R (2015). Crustal structure and deformation under the Longmenshan and its surroundings revealed by receiver function data. Phys. Earth Planet. Inter..

[44] Zhao GZ, Unsworth MJ, Zhan Y, Wang LF (2012). Crustal structure and rheology of the Longmenshan and Wenchuan Mw 7.9 earthquake epicentral area from magnetotelluric data. Geology.

[45] Pei S (2010). Three-dimensional seismic velocity structure across the 2008 Wenchuan Ms 8.0 earthquake, Sichuan, China. Tectonophysics.

[46] Wang Z, Huang R, Pei S (2014). Crustal deformation along the Longmen-Shan fault zone and its implications for seismogenesis. Tectonophysics.

[47] Li ZW, Ni SD, Roecker S (2014). Interstation Pg and Sg differential traveltime tomography in the northeastern margin of the Tibetan plateau: Implications for spatial extent of crustal flow and segmentation of the Longmenshan fault zone. Phys. Earth Planet. Inter..

[48] Li C, van der Hilst R, Meltzer AS, Engdahl ER (2008). Subduction of the Indian lithosphere beneath the Tibetan Plateau and Burma. Earth Planet. Sci. Lett..

[49] Wang Z, Zhao D, Wang J (2010). Deep structure and seismogenesis of the north-south seismic zone in southwest China. J. Geophys. Res..

[50] Lei JS, Zhao DP (2006). Global P-wave tomography: on the effect of various mantle and core phases. Phys. Earth Planet. Inter..

[51] Watson MP, Hayward AB, Parkinson DN, Zhang ZM (1987). Plate tectonic history, basin development and petroleum source rock deposition onshore China. Mar. Petrol. Geol..

[52] Liu S (2001). Study on tectonic events in the LongmenMountains-western Sichuan foreland basin, Songpan-Gangze, China. J. Chengdu univ. Technol..

[53] Weislogel AL (2008). Tectonostratigraphic and geochronologic constraints on evolution of the northeast Paleotethys from the Songpan-Ganzi complex, central China. Tectonophysics.

[54] Billerot A, Duchene S, Vanderhaeghe O, de Sigoyer J (2017). Gneiss domes of the Danba metamorphic complex, Songpan Ganze, eastern Tibet. J. Asian Earth Sci..

[55] Harrowfield MJ, Wilson CJL (2005). Indosinian deformation of the Songpan Garze Fold Belt, northeast Tibetan Plateau. J. Struct. Geol..

[56] Huang MH, Buick IS, Hou LW (2003). Tectonometamorphic evolution of the Eastern Tibet plateau: evidence from the Central Songpan-Garze orogenic belt, Western China. J. Petrol..

[57] Huang ZC (2015). Mantle structure and dynamics beneath SE Tibet revealed by new seismic images. Earth Planet. Sci. Lett..

[58] Yang T, Wu JP, Wang WL (2014). Complex Structure beneath the Southeastern Tibetan Plateau from Teleseismic P-Wave Tomography. Bull. Seismol. Soc. Am..

[59] Chen M, Niu F, Liu Q, Tromp J, Zheng X (2015). Multiparameter adjoint tomography of the crust and upper mantle beneath East Asia: 1. Model construction and comparisons. J. Geophys. Res..

[60] Bao XW, Song XD, Li JT (2015). High-resolution lithospheric structure beneath Mainland China from ambient noise and earthquake surface-wave tomography. Earth Planet. Sci. Lett..

[61] Wei W, Xu JD, Zhao D, Shi YL (2012). East Asia mantle tomography: New insight into plate subduction and intraplate volcanism. J. Asian Earth Sci..

[62] Li C, van der Hilst RD, Toksöz MN (2006). Constraining P-wave velocity variations in the upper mantle beneath Southeast Asia. Phys. Earth Planet. Inter..

[63] Wei W, Zhao D, Xu J, Zhou B, Shi YL (2016). Depth variations of P-wave azimuthal anisotropy beneath Mainland China. Scientific Reports.

[64] Shen W (2016). A seismic reference model for the crust and uppermost mantle beneath China from surface wave dispersion. Geophy. J. Int..

[65] Xin Hailiang, Zhang Haijiang, Kang Min, He Rizheng, Gao Lei, Gao Ji (2018). High‐Resolution Lithospheric Velocity Structure of Continental China by Double‐Difference Seismic Travel‐Time Tomography. Seismological Research Letters.

[66] Teng, J., Zhang, Z. & Bai, W. Physics of the Lithosphere (ed. Teng, J., Zhang, Z. & Bai, W.) 990 (Chinese Sciences Press, Beijing 2003).

[67] Cook FA, Velden A, Hall KW, Roberts B (1999). Frozen subduction in Canadas Northwest Territories: lithoprobe deep lithospheric reflection profiling of the western Canadian Shield. Tectonics.

[68] Balling N (2000). Deep seismic reflection evidence for ancient subduction and collision zones within the continental lithosphere of northwestern Europe. Tectonophysics.

[69] Svenningsen L (2007). Crustal root beneath the highlands of southern Norway resolved by teleseismic receiver functions. Geophys. J. Inter..

[70] Zhai MG, Fan QC, Zhang HF, Sui JL, Shao JA (2007). Lower crustal processes leading to Mesozoic lithospheric thinning beneath eastern North China: Underplating, replacement and delamination. Lithos.

[71] He CS, Santosh M, Dong SW (2015). Continental dynamics of Eastern China: insights from tectonic history and receiver function analysis. Earth-Sci. Rev..

[72] Zhao D, Hasegawa A, Horiuchi S (1992). Tomographic imaging of P- and S-wave velocity structure beneath northeastern Japan. J. Geophys. Res..

[73] Xu YG, He B, Chung SL, Menzies MA, Frey FA (2004). Geologic, geochemical, and geophysical consequences of plume involvement in the Emeishan flood-basalt province. Geology.

[74] Shen Y (2002). Seismic evidence for a tilted mantle plume and north-south mantle flow beneath Iceland. Earth Planet. Sci. Lett..

[75] Chen M (2016). Lithospheric foundering and underthrusting imaged beneath Tibet. Nature Communications.

[76] Replumaz A, Negredo AM, Guillot S, Villaseñor A (2010). Multiple episodes of continental subduction during India/Asia convergence: insight from seismic tomography and tectonic reconstruction. Tectonophysics..

[77] Van der Voo R, Spakman W, Bijwaard H (1999). Mesozoic subducted slabs under Siberia. Nature.

[78] Ueda K, Gerya TV, Burg JP (2012). Delamination in collisional orogens: Thermomechanical modeling. J. Geophys. Res..

[79] Zhang HF (2007). A-type granite and adakitic magmatism association in Songpan-Garze fold belt, eastern Tibetan Plateau: Implicaiton for lithospheric delamination. Lithos.

[80] Chen W (2017). Combined paleomagnetic and geochronological study on Cretaceous strata of the Qiangtang terrane, central Tibet. Gondwana Res..

[81] Deschamps F (2017). Coeval mantle-derived and crust-derived magmas inside the orogenic crust exemplified by two neighbouring plutons of the Songpan Ganze accretionary-orogenic wedge (SW China). J. Petrol..

[82] Huang MH, Maas R, Buick IS, Williams I (2003). Crustal response to continental collisions between the Tibet, Indian, South China and North China blocks: geochronological constraints from the Songpan-Garze Orogenic Belt, Western China. J. Metamorph. Geol..

[83] de Sigoyer J, Vanderhaeghe O, Duchêne S, Billerot A (2014). Generation and emplacement of Triassic granitoids within the Songpan Ganze accretionary-orogenic wedge in a context of slab retreat accommodated by tear faulting, Eastern Tibetan plateau, China. J. Asian Earth Sci..

[84] Robert A (2010). Structural and thermal characters of the Longmen Shan (Sichuan, China). Tectonophysics.

[85] Kay RW, Kay SM (1993). Delamination and delamination magmatism. Tectonophysics.

[86] He CS, Santosh M (2017). Mantle roots of the Emeishan plume: an evaluation based on telesismic P-wave tomography. Solid Earth.

[87] Royden L (1996). Coupling and decoupling of crust and mantle in convergent orogens: Implications for strain partitioning in the crust. J. Geophys. Res..

[88] Klemperer SL (2006). Crustal flow in Tibet: A review of geophysical evidence for the physical state of Tibetan lithosphere, in Channel Flow, Ductile Extrusion and Exhumation of Lower Mid-Crust in Continental Collision Zones. Geol. Soc. Spec. Publ..

[89] Vanderhaeghe O, Teyssier C (2001). Partial melting and flow of orogens. Tectonophysics.

[90] Xu Q, Zhao J, Yuan X, Liu H, Pei S (2015). Mapping crustal structure beneath southern Tibet: Seismic evidence for continental crustal underthrusting. Gondwana Res..

[91] England PC, Thompson A (1986). Some thermal and tectonic models for crustal melting in continental collision zones. Geol. Soc. London Spec. Pub..

[92] Vanderhaeghe O, Medvedev S, Fullsack P, Beaumont C, Jamieson RA (2003). Evolution of orogenic wedges and continental plateaux: insights from crustal thermal–mechanical models overlying subducting mantle lithosphere. Geophys. J. Int..

[93] Nelson K (1996). Partially molten middle crust beneath southern Tibet: Synthesis of project INDEPTH results. Science.

[94] Xu Q, Zhao J, Pei S, Liu H (2013). Distinct lateral contrast of the crustal and upper mantle structure beneath northeast Tibetan plateau from receiver function analysis. Phys. Earth Planet. Inter..

[95] Chang CP (2012). Influence of the pre-existing Xiaoyudong salient in surface rupture distribution of the Mw 7.9 Wenchuan earthquake, China. Tectonophysics.

[96] Molnar P, Tapponnier P (1975). Cenozoic Tectonics of Asia: effects of a continental collision. Science.

[97] Lev E, Long MD, van der Hils RD (2006). Seismic anisotropy in Eastern Tibet from shear wave splitting reveals changes in lithospheric deformation. Earth Planet. Sci. Lett..

[98] Royden LH, Burchfiel BC, van der Hilst RD (2008). The geological evolution of the Tibetan plateau. Science.

[99] Lin A (2011). Co-seismic strike-slip surface rupture and displacement produced by the 2010 MW 6.9 Yushu earthquake, China, and implications for Tibetan tectonics. J Geodyn..

[100] Klinger Y (2005). High resolution satellite imagery mapping of the surface rupture and slip distribution of the M-W similar to 7.8, 14 November 2001 Kokoxili Earthquake, Kunlun Fault, northern Tibet, China. Bull. Seismol. Soc. Am..

[101] Peltzer G, Crampé F, King G (1999). Evidence of Nonlinear Elasticity of the Crust from the Mw 7.6 Manyi (Tibet) Earthquake. Science.

[102] Xu X, Yu G, Klinger Y, Tapponnier P, Van der Woerd J (2006). Re-evaluation of surface rupture parameters and faulting segmentation of the 2001 Kunlunshan earthquake (Mw7.8), Northern Tibetan Plateau, China. J. Geophy. Res..

[103] Wang H (2014). Crustal structure and Moho geometry of the northeastern Tibetan plateau as revealed by SinoProbe-02 deep seismic-reflection profiling. Tectonophysics.

[104] Luna LM, Hetland EA (2013). Regional stresses inferred from coseismic slip models of the 2008 Mw 7.9 Wenchuan, China, earthquake. Tectonophysics.

[105] Zheng XF, Yao ZX, Liang JH, Zheng J (2010). The role played and opportunities provided by IGP DMC of China National Seismic Network in Wenchuan earthquake disaster relief and researches. Bull. Seismol. Soc. Am..

[106] VanDecar, C. & Crosson, S. Determination of teleseismic relative phase arrival times using multi-channel cross-correlation and least squares. *Bull*. *Seismol*. *Soc*. *Am*. **80**, 150–169, http://www.bssaonline.org/content/80/1/150.short (1990).

[107] Paige CC, Saunders MA (1982). LSQR: an algorithm for sparse linear equations and spare least squares. ACMTrans. Math. Softw..

[108] Kennett B, Engdahl E (1991). Traveltimes for global earthquake location and phase identification. Geophys. J. Int..

[109] Laske G, Masters G, Ma Z, Pasyanos ME (2012). CRUST1.0: an updated global model of Earth’s Crust. Geophys. Res. Abstracts.

[110] Jiang GM (2015). Mantle dynamics and Cretaceous magmatism in east-central China: Insight from teleseismic tomograms. Tectonophysics.

[111] Zhao DP, Lei JS, Inoue T, Yamada Y, Gao S (2006). Deep structure and origin of the Baikal rift zone. Earth Planet. Sci. Lett..

[112] Hansen P (1992). Analysis of discrete ill-posed problems by means of the L-curve. SIAM Rev..

[113] Boschi L, Becker T, Soldati G, Dziewonski AM (2006). On the relevance of Born theory in global seismic tomography. Geophys. Res. Lett..

[114] Lei JS, Zhao DP, Steinberger B, Shen FL, Li ZX (2009). New seismic constraints on the upper mantle structure of the Hainan plume. Phys. Earth Planet. Inter..

