# Supplementary material for: Lithospheric delamination and upwelling asthenosphere in the Longmenshan area: insight from teleseismic P-wave tomography
Source: Sci Rep. 2019 May 6;9:6967. doi: 10.1038/s41598-019-43476-0 (PMC6503211; doi:10.1038/s41598-019-43476-0)
Supplement: Supplementary file 1 — Supplementary Information [file 41598_2019_43476_MOESM1_ESM.docx]

Lithospheric delamination and upwelling asthenosphere in the Longmenshan area: insight from teleseismic P-wave tomography

Chuansong He^1^[[1]](#footnote-1)^*^, Shuwen Dong ^2^[[2]](#footnote-2)^*^, Yanghua Wang ^3^

*1 Institute of Geophysics, CEA, Beijing 100081, China*

*2 State Key Laboratory for Mineral Deposits Research, Nanjing University, Nanjing 210093, China*

*3 Department of Earth Science and Engineering, Imperial College London, South Kensington, London SW7 2BP, UK*


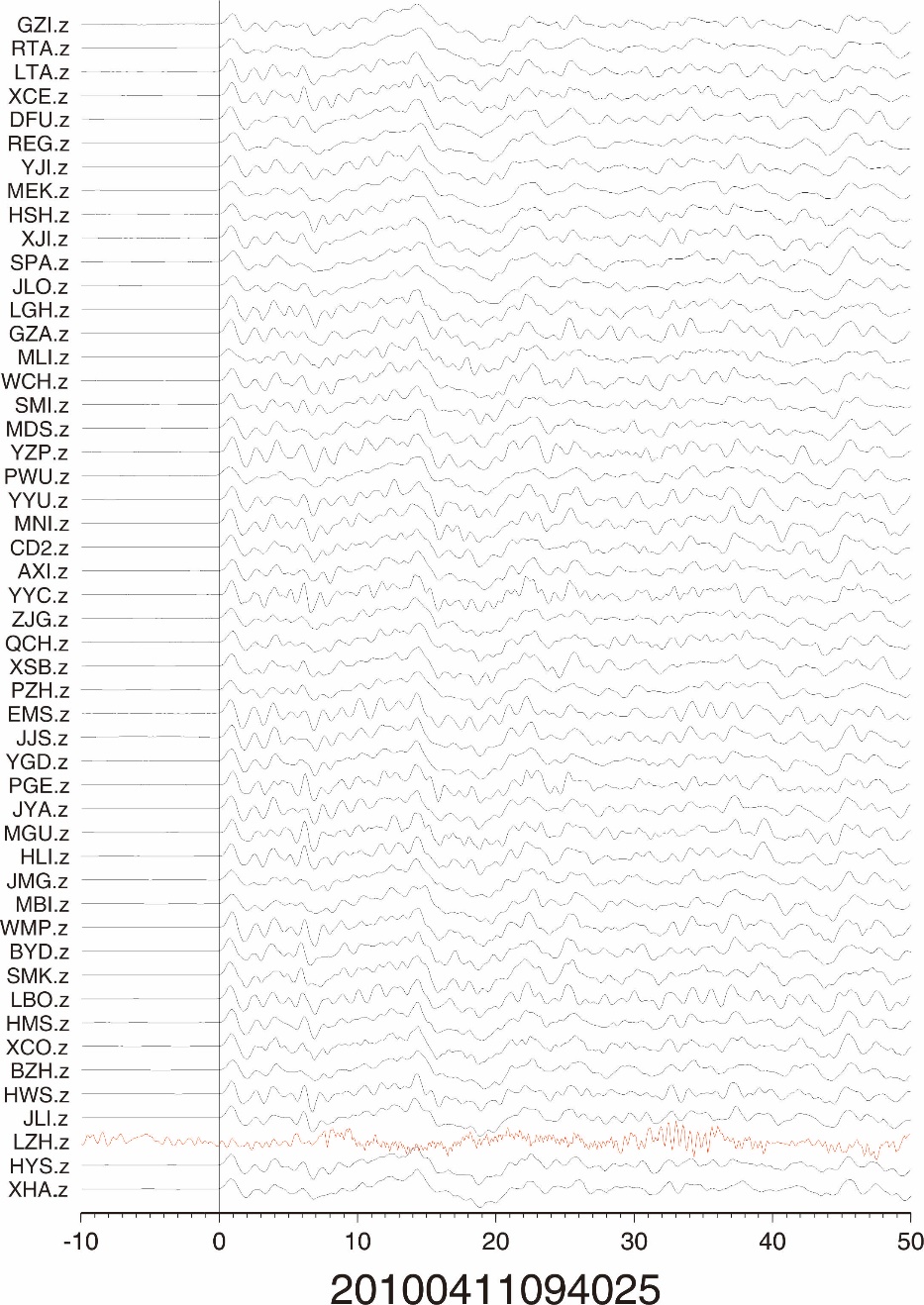


Fig. S1 Example: P-wave arrivals are picked from the cut and filtered seismograms of seismic events using the time cross correlation method. Earthquake event: 20100411094025; waveform: vertical channel record of seismic station, red waveform: abandoned record (The figure is generated using Generic Mapping Tool (http://gmt.soest.hawaii.edu/) by Chuansong He).


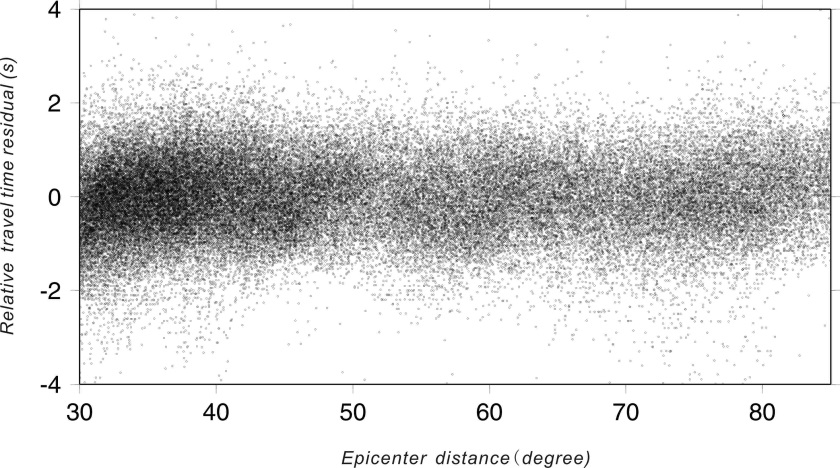


Fig. S2 Distribution of relative travel-time residuals. The range of -2.5 s to +2.5 s of the relative travel-time residuals are used in the tomographic inversion (The figure is generated using Generic Mapping Tool (http://gmt.soest.hawaii.edu/) by Chuansong He).


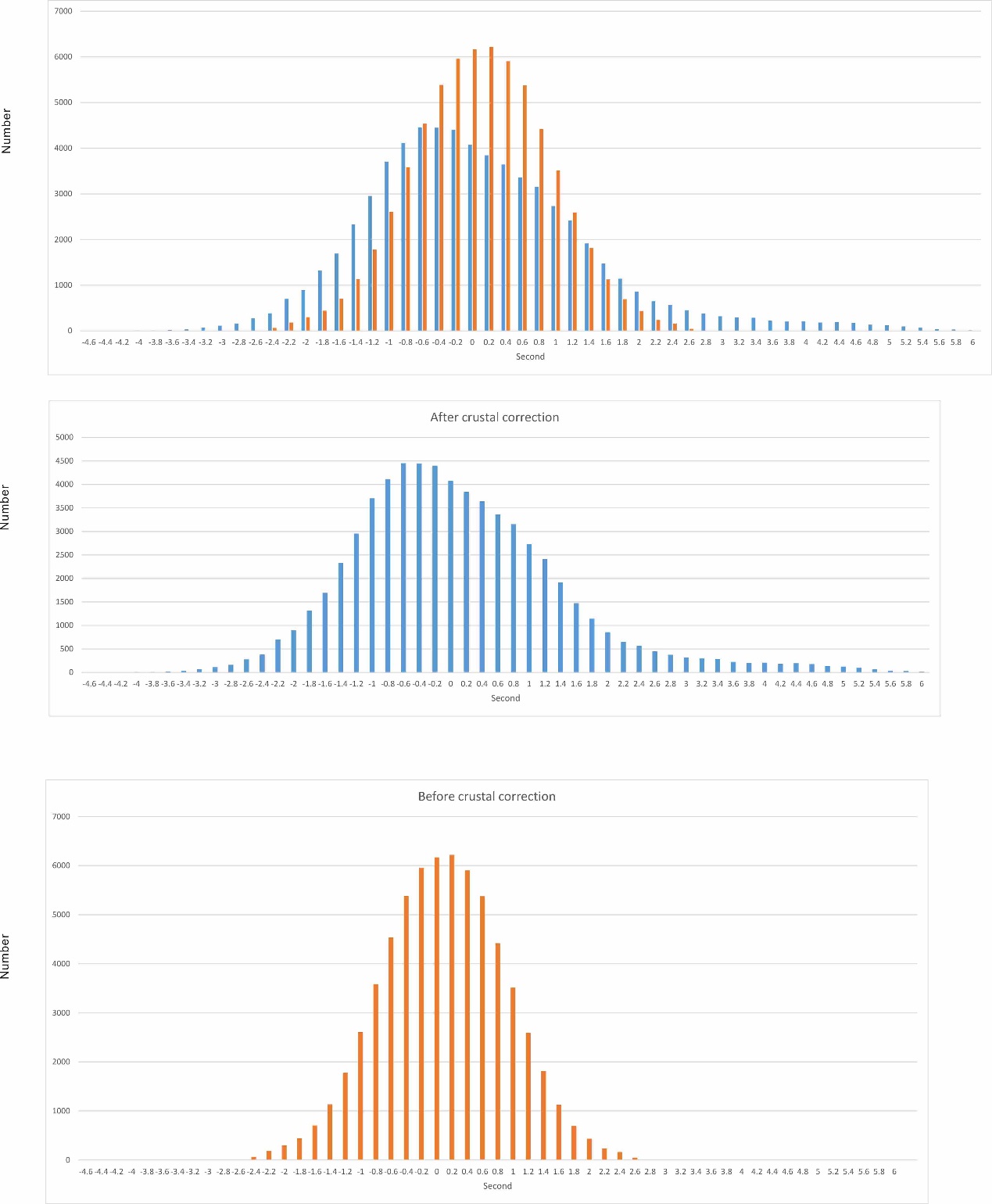


Fig. S3 Distribution of relative travel-time residuals before and after crustal correction.


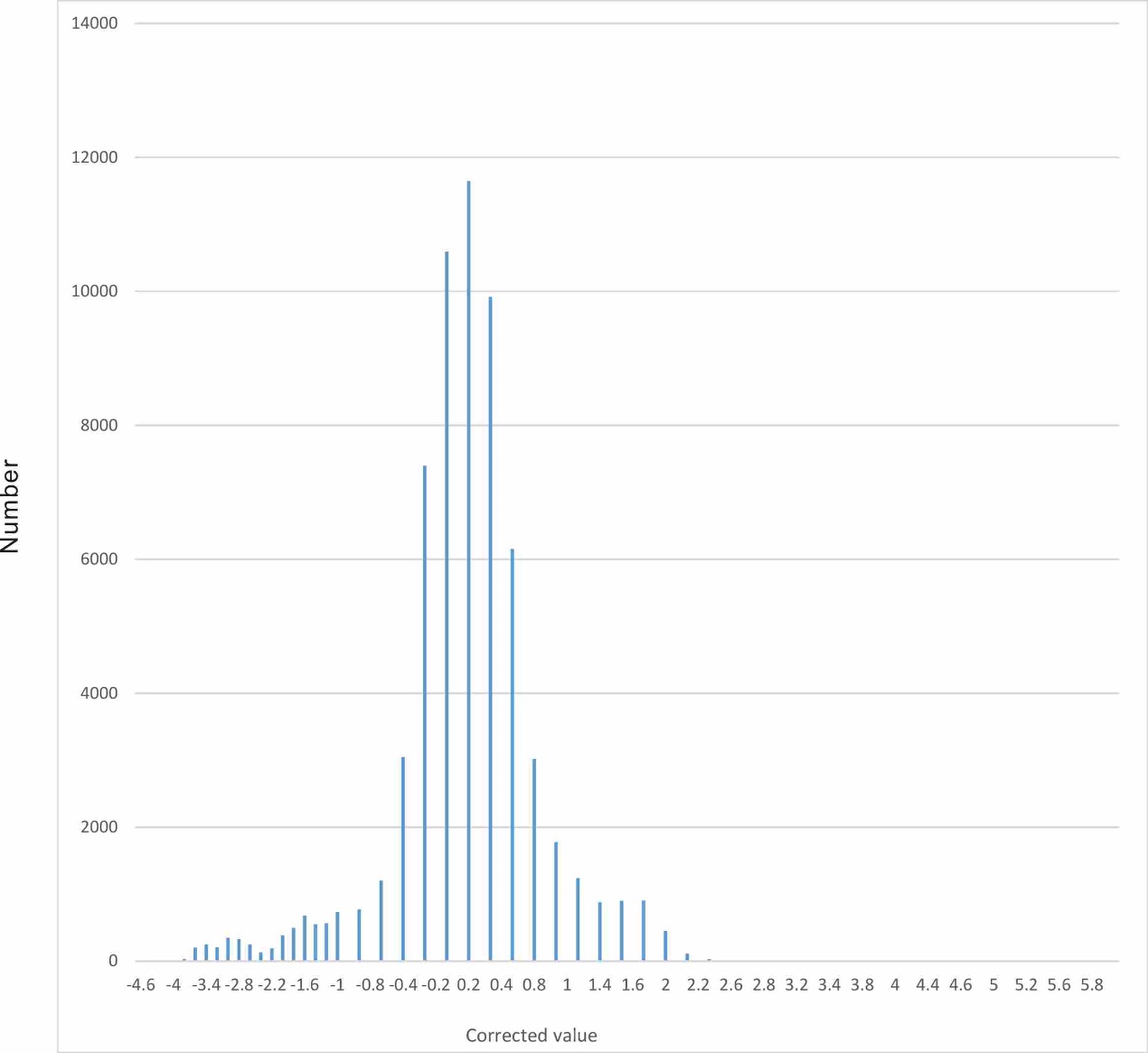


Fig. S4 Distribution of corrected value of crustal correction.


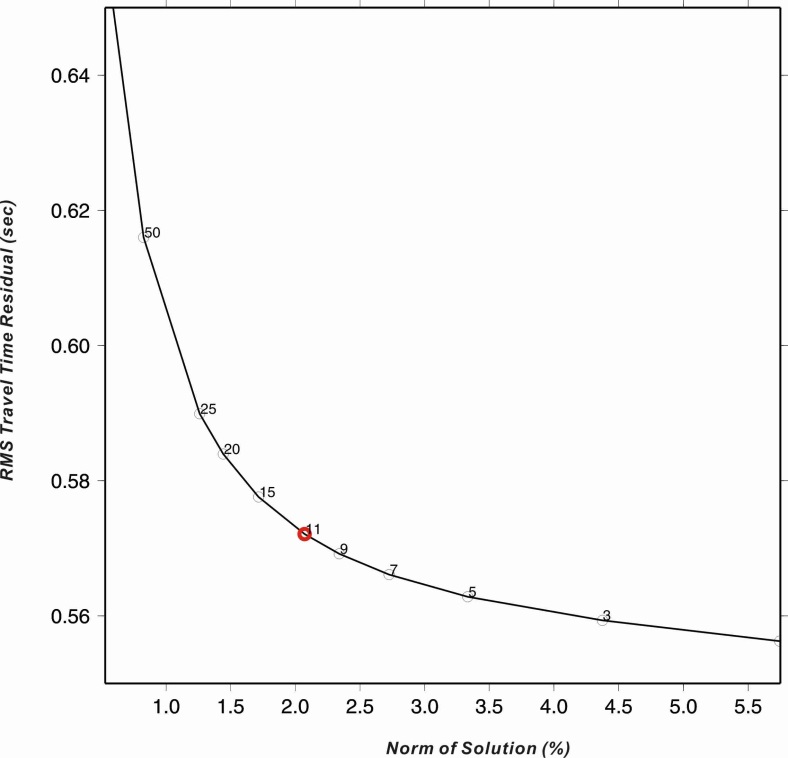


Fig. S5 The damping parameter (11) taken to invert final solution model (red circle) after a series inversion test. RMS travel time residual is about 0.57209 s (The figure is generated using Generic Mapping Tool (http://gmt.soest.hawaii.edu/) by Chuansong He).


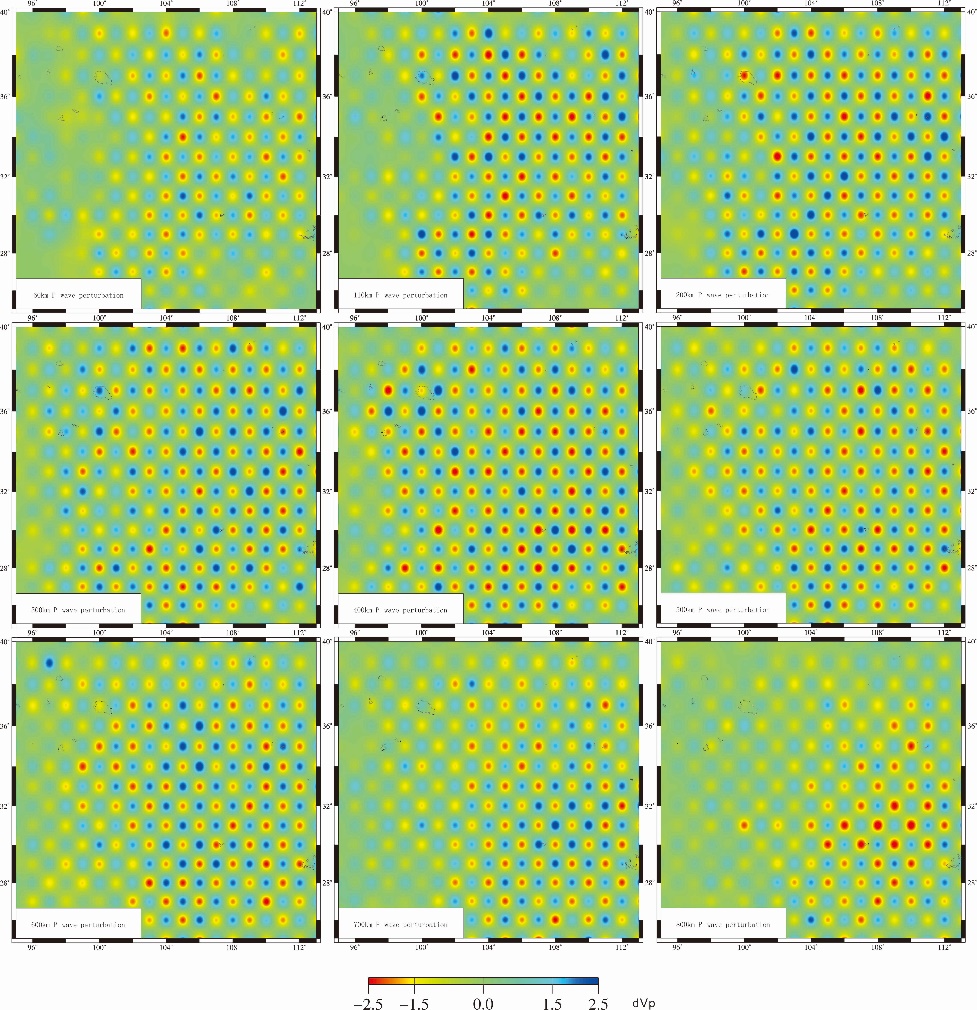


Fig. S6 Map views of CRT results at depths of 60, 110, 200, 300, 400, 500, 600, 700 and 800 km. The model was run using the same raypaths and the same damping parameter as the main inversion (The figure is generated using Generic Mapping Tool (http://gmt.soest.hawaii.edu/) by Chuansong He).

1. * Corresponding author. *Email address*: hechuansong@aliyun.com [↑](#footnote-ref-1)
2. * Corresponding author. *Email address*: swdong@cags.ac.cn [↑](#footnote-ref-2)
